# Trends in Jewish Young Adult Experiences and Perceptions of Antisemitism in America from 2017 to 2019

**DOI:** 10.1007/s12397-021-09354-6

**Published:** 2021-02-15

**Authors:** Graham Wright, Sasha Volodarsky, Shahar Hecht, Leonard Saxe

**Affiliations:** 1 Brandeis University, 415 South Street, MS 014, Waltham, MA 02453-2728 USA; 2 Northeastern University, Boston, USA

**Keywords:** Antisemitism, American Jews, Public opinion, College students, Young adults

## Abstract

Since 2016, a series of horrific acts motivated by antisemitism appear to have caused a fundamental shift in the prevalence of antisemitism in the United States. Little is known, however, about how the events during this time have affected the day-to-day experiences and concerns of American Jews. Using repeated cross-sectional data from surveys of Jewish young adults who applied to Birthright Israel, this paper analyzes recent trends in Jewish young adults’ experiences and perceptions of antisemitism. Despite the high-profile incidents during this period, there is no evidence of a major increase in experiences of antisemitic harassment among Jewish young adults—either on or off campus—between 2017 and 2019. At the same time, data show a substantial increase in concerns among young Jews about antisemitism in the United States and on college campuses, with concerns about antisemitism in the United States (but not on campus) being especially concentrated among liberal Jews. These results suggest that Jewish concerns about antisemitism are linked to broader views about the climate for marginalized populations in the United States. They also point to growing Jewish anxieties over violence, safety, and acceptance in the United States. In an era of widely disseminated antisemitic conspiracy theories, even young Jews who have no first-hand experience of antisemitic harassment have become concerned.

**Supplementary Information:**

The online version of this article (10.1007/s12397-021-09354-6) contains supplementary material, which is available to authorized users.

Since 2016, a series of violent, widely reported incidents have ignited concerns that antisemitism in the United States is reemerging after decades in which it appeared to be in decline. In August 2017, the “Unite the Right” rally in Charlottesville, Virginia, saw white nationalists chanting “Jews will not replace us!,” and the death of a counter-protester (Gray 2017). In October 2018, a gunman obsessed with an antisemitic conspiracy theory murdered 11 Jews in the Tree of Life synagogue in Pittsburgh, Pennsylvania (Turkewitz and Roose 2018). In April of 2019, another gunman attacked a Chabad synagogue in Poway, California, killing one congregant (Van Sant and Doubek 2019). The same period also witnessed increased reports of less severe antisemitic incidents, especially on college campuses (AMCHA Initiative 2018; The Center on Extremism 2017). These incidents seem to suggest that there has been a fundamental shift in the nature and prevalence of antisemitism in America, portending a return to an era where physical intimidation of Jews was routine and antisemitic discrimination was explicit and pervasive.

Nevertheless, little is known about how recent events have affected the day-to-day experiences and concerns of American Jews. This question is of particular interest with respect to younger Jews, who grew up in an era where antisemitism appeared dormant, and for whom it may be shocking to see the sort of virulent antisemitism they have only read about in history books. Furthermore, many of these young Jews find themselves on college campuses, at the center of what some have called hotbeds of antisemitism and anti-Israel activity (Bauer-Wolf 2018; Speyer 2018). Have the events of 2017–19 been accompanied by an increase in the prevalence of antisemitic harassment against Jewish young adults, either on campus or off? How have the concerns of young adults about antisemitism changed during a period where its existence has again become front-page news? How are these concerns influenced by what appears to be a growing political divide over the cause and locus of resurgent antisemitism in America? In this paper we seek answers to these questions using repeated cross-sectional data from a sample of Jewish young adults. Our goal is to document and analyze trends in Jewish young adults’ experiences and perceptions of antisemitism between 2017 and 2019.

## Antisemitism: Here and Now?

The second half of the twentieth century witnessed a dramatic decline in the expression of antisemitic attitudes among the American public (Smith and Schapiro 2018). By the second decade of the new millennium, Jews had shaken off centuries of discrimination to emerge as the most positively viewed religious group in America (Pew Research Center 2017). In recent years, however, a series of incidents, including the shootings in Pittsburgh and Poway, have prompted warnings that a previously quiescent stratum of antisemitism lurking beneath American society has now resurfaced, in an even more virulent form (Lipstadt 2019; Reich 2020; Paul 2019). Some commentators warn of a revival of antisemitic attitudes and stereotypes by President Trump and his supporters in the “alt-right” movement (Weisman 2018; Moshin 2018; Golinkin 2019). Others claim that a “crisis” of antisemitism is brewing on US college campuses (Marcus 2016; Bauer-Wolf 2018; Flayton 2019), especially in relation to the Boycotts, Divestments and Sanctions (BDS) movement (AMCHA Initiative 2018; Speyer 2018; Marcus 2015). Commentators have raised special concern over the impact of these events on Jewish young adults, many of whom grew up without ever being “scared to be Jewish” (Zauzmer 2018). Insulated from first- or even second-hand experience with discrimination, young adult Jews who “feel no constraining tug from the Jewish past” (Kelner 2019) may have particular difficulty coming to terms with an era marked by violent antisemitism of the sort they had only read about in history books.

Documenting changes in the nature and prevalence of antisemitism during this period is a challenge. Since 2016, there have been relatively few efforts to assess whether antisemitic attitudes have become more prevalent among the American public (Smith and Schapiro 2018). One of the only recent sources of data, the Anti-Defamation League’s (ADL) long-running survey on antisemitic attitudes, suggests that between 2015 and 2019, the proportion of Americans who agree with various antisemitic statements has declined slightly (Anti-Defamation League 2020). But the utility of these data may be limited, since research suggests that many Americans harbor “hidden” or “latent” antisemitism that is difficult or impossible to measure with traditional survey questions (Cohen et al. 2009).

Even before the events of Charlottesville, Pittsburgh, and Poway, efforts to identify trends in US antisemitism had already shifted to the cataloging of antisemitic *incidents.* Groups like the AMCHA Initiative and the Anti-Defamation League (ADL) have created reports and data sets cataloging various forms of antisemitic activity, including antisemitic graffiti and vandalism, antisemitic posts on social media, and threats to Jewish institutions (e.g., AMCHA Initiative 2018; The Center on Extremism 2017)*.* When aggregated, these incident catalogs provide some evidence of an increase in US antisemitism since 2016, although their reliability is hampered by several methodological challenges (Smith and Schapiro 2018).

Nevertheless, catalogs of antisemitic incidents—no matter how accurate—do not provide a reliable measure of the overall prevalence of antisemitism in US society or its impact on the day-to-day lives of American Jews. First, many of the more common forms of antisemitic harassment, either in person or online, are unlikely to generate reportable “incidents.” Second, a particular incident, such as the defacement of a wall with antisemitic graffiti, can be cataloged as an antisemitic incident even if few Jews are aware of it. Third, increases in antisemitic incidents may actually be a result of the actions of a small number of individuals. In 2017, the ADL’s finding of a dramatic spike in antisemitic incidents was partly due to the actions of a single Israeli-American teenager who made hundreds of threatening calls to Jewish institutions during this period (Smith and Schapiro 2018).

Therefore, an increase in the number of reported antisemitic incidents may not indicate an increase in the level of antisemitic harassment experienced by Jews. Finally, the catalogs of antisemitic incidents cannot measure how Jews *feel* about antisemitism in America. Do American Jews see the events of 2017–19 as isolated occurrences or as harbingers of a new wave of anti-Jewish violence and bigotry? These challenges suggest the need for a more robust analysis of recent US trends in antisemitism, both on and off campus. Instead of focusing on incidents, such an analysis would explore changes in Jewish experiences *and* perceptions of antisemitism between 2017 and 2019.

## American Jewish Experiences and Perceptions of Antisemitism

Past research has recognized the importance of distinguishing between Jewish *experiences* and *perceptions* of antisemitism (Rebhun 2014). Although these phenomena are sometimes treated as different measures of the overall level of antisemitism in a society (e.g., Smith and Schapiro 2018; Kremelberg and Dashefsky 2016) and are both important, they should be distinguished. Experiences of antisemitism serve as a measure of the prevalence of antisemitic harassment, while perceptions of antisemitism reflect Jewish concerns and anxieties about vulnerability and safety in different societal contexts. The events of 2017–19 may have impacted these two phenomena in different ways and to different degrees.

Tracking reports of personal experiences of antisemitism among comparable samples of Jews between 2017 and 2019 can help to determine whether it has become more (or less) common for individual Jews to experience antisemitic harassment or discrimination during this period. But trends in antisemitic harassment may be substantially different for Jews in different situations. Thus, for example, if warnings of a growing crisis of antisemitism on the college campus (Speyer 2018; Bauer-Wolf 2018) are accurate, Jewish undergraduates may have experienced a steeper increase in antisemitic harassment than similar Jewish young adults who are not undergraduates. At the same time, if the crisis of antisemitism on campus has been, as some claim, “manufactured” (Samel 2020), there may be little or no difference between undergraduates and non-undergraduate Jewish young adults in rates of antisemitic harassment. To investigate this question, it is important to ensure that differences in experiences of antisemitism between undergraduates and non-undergraduates are not confounded by other intervening variables. For example, past research has found that Jews with stronger religious backgrounds are more likely to experience antisemitic harassment (Kremelberg and Dashefsky 2016; Rebhun 2014). If Jewish undergraduates experience higher (or lower) rates of antisemitism compared to other young adult Jews, this may reflect the fact that Jewish undergraduates are more likely to have a stronger religious background or some other intervening relationship.

Similarly, perceptions of antisemitism may be exacerbated by personal experiences of antisemitism (Rebhun 2014). But even without personal experiences of antisemitic harassment, Jewish concerns about antisemitism may reflect anxieties about their continued vulnerability in American society (Berenbaum 2016). Research on other forms of discrimination finds that *perceived* discrimination can itself contribute to detrimental health outcomes (Beccerra et al. 2013; Pachter et al. 2010; Pascoe and Richman 2009) and feelings of social alienation (Cabrera and Nora 1994) and can undermine relationships between minority groups in American society (Pettigrew 1998).

As such, researchers have long been interested in Jewish concerns about antisemitism in the US (Kim 1995; Tobin and Sassler 1988) and have sought to understand why, despite the apparent decline in antisemitic attitudes among the US public, a majority of American Jews perceive antisemitism to be a “serious problem” (Cohen 2010, 2018). This work finds that Jewish concerns about antisemitism are strongly influenced by media reports of antisemitism (Cohen 2010) as well as perceptions of discrimination against *other* minority groups (Cohen 2018), suggesting that many Jews feel that their fate is connected to that of other marginalized populations in the US. Understanding the perceptions of Jewish “millennials” and “Gen Z-ers,” who hold dramatically different views on America’s treatment of minority groups compared to previous generations (Parker and Igielnik 2020), and who likely had relatively little firsthand exposure to antisemitism prior to 2016, is particularly important.

Nevertheless, it should not be assumed that there has been a single, uniform trend in concerns over antisemitism during this period among Jewish young adults. Concerns over antisemitism may be greater in some contexts than others, and may be dramatically different across different groups of Jews. Given concerns about antisemitism on college campuses, many young adult Jews—whether they are students or not—may feel that antisemitism is not a serious problem in the United States in general, but that it *is* a serious problem on campus. Conversely, others may still be pessimistic about the current climate for antisemitism in the United States in general, but dismiss concerns about antisemitism on the college campus. Concerns about antisemitism in both of these contexts may be strongly influenced, albeit in different ways, by a factor that has not yet been deeply investigated as a predictor of Jewish attitudes towards antisemitism: political ideology.

## Antisemitism on the Right and Left

Antisemitism has long been seen as connected to “right-wing authoritarianism” (Adorno et al. 1950) and other forms of racist and ethno-nationalist bigotry (Dunbar 1995; Dunbar and Simonova 2003; Reisigl and Wodak 2001). Cohen (2018) demonstrates that this association exists in Jewish public opinion as well. Not surprisingly, many scholars and media commentators have connected the apparent rise in antisemitism to broader increases in racism and nationalism during the Trump era (Moshin 2018; Weisman 2018), especially in light of President Trump’s controversial claim that there were “some very fine people on both sides” of the Unite the Right rally in Charlottesville (Gray 2017).

But the same period has also seen growing apprehension over a “new antisemitism” that is closely connected to (predominantly) left-wing criticism of Israel (Cohen et al. 2009; Judaken 2008). Because allegations of “left” antisemitism are often connected to the activities of groups like BDS that operate on campus (Marcus 2016), conservative discourses on antisemitism often highlight the college campus as the locus of antisemitism (French 2019). President Trump himself has attempted to frame the issue of antisemitism in this way by signing a 2019 executive order aimed at “defend[ing] Jewish students” by encouraging university administrators to treat certain forms of Israel criticism as antisemitic discrimination (Kushner 2019).

Although these two narratives about antisemitism are not mutually exclusive, we hypothesize that a political divide exists in how Jews perceive antisemitism in the United States. Liberal Jews, because they are more likely to see antisemitism as reflective of a broader increase in right-wing bigotry and prejudice during the Trump administration, are likely to express higher levels of concern about antisemitism in the US in general, compared to their conservative peers. In contrast, conservative Jews, because they are more likely to see “new antisemitism” as driven by left-wing criticism of Israel on campus, are likely to express higher levels of concern about antisemitism *on the college campus* compared to their liberal peers.

To test these hypotheses and understand the ways in which the events of 2017–19 have affected Jewish young adults, we use repeated cross-sectional data to analyze changes in Jewish young adults’ experiences and perceptions of antisemitism over time.^1^ We first analyze trends in personal experiences with antisemitism among undergraduates and non-undergraduates to determine whether there has been an increase in the prevalence of antisemitic harassment, either on or off campus. We then track Jewish young adult concerns about antisemitism on the college campus, and with regard to the United States more broadly. Finally, we analyze the extent to which Jewish concerns about antisemitism on campus or in the United States are influenced by political ideology in the manner hypothesized above.

## Data and Methods

Data for these analyses come from a series of surveys of US applicants to the Birthright Israel program, which offers free trips to Israel to Jewish young adults between the ages of 18 and 27 (Saxe and Chazan 2008). The surveys were of applicants to summer trips in 2017, 2018, and 2019.^2^ In each of these three years, those who applied to go on a Birthright trip during the summer were surveyed in March or April (approximately three months before the trip) and again in November (approximately four months after the trip). Summer 2018 applicants were also surveyed a third time in November 2019 (approximately 16 months after their trip). Data are available from six separate time points during the three-year period. In all cases, surveys were sent to *all* eligible Birthright applicants, including those who did not actually go on the trips.^3^ Table 1 shows the number of respondents for each of these six time points and the overall response rate of each survey.

**Table 1:** Achieved sample size and response rate for each time point

	*N*	Response rate (AAPOR RR2)
March 2017 (Pre-trip surveys of summer 2017 applicants)	6904	23%
Nov 2017 (Post-trip surveys of summer 2017 applicants)	4607	15%
Apr 2018 (Pre-trip surveys of summer 2018 applicants)	8206	31%
Nov 2018 (Post-trip surveys of summer 2018 applicants)	2995	13%
Apr 2019 (Pre-trip surveys of summer 2019 applicants)	6177	25%
Nov 2019 (Post-trip surveys of summer 2019 applicants and follow-up surveys of summer 2018 applicants)	5057	13%, 10%
Total	32,596	

Achieved sample sizes and response rates include partial respondents. AAPOR RR2: American Association for Public Opinion Research Response Rate 2

The sample for these analyses is not fully representative of all Jewish young adults, insofar as it includes only those between the ages of 18 and 27, and only those who applied to Birthright Israel.^4^ Although these analyses cannot produce reliable estimates of the *overall* percentage of Jewish young adults who experienced or were concerned about antisemitism during this period, the study explores *changes* in perceptions and experiences of antisemitism and the extent to which these changes differ among subpopulations (e.g., undergraduate students or liberals).

One threat to the validity of comparisons involving the use of multiple cross-sectional surveys is the possibility that demographic characteristics of respondents have changed over time, either due to changes in nonresponse bias in each survey or to changes in the underlying applicant pool. To address this challenge, we calculated post-stratification weights that adjusted respondents’ characteristics from all time points to the background characteristics of applicants to the summer 2017 trips, using information collected during the Birthright application process.^5^ This adjustment helps to ensure that changes in the proportion of applicants who experienced or perceived antisemitism in each time period are not due to changes in the sample composition.

Respondents to all six surveys were asked whether, in the past three months, they had been insulted or harassed in person, insulted or harassed on social media, or physically attacked *because they were Jewish*. We measure experiences of antisemitism with a binary variable that denotes respondents who reported any of these three experiences. All respondents, regardless of whether they were college students, were also asked how concerned they were about antisemitism in the United States, and on college campuses,^6^ with response options of “not at all,” “a little,” “somewhat,” and “very much.” We measured concern about antisemitism in each of these contexts with binary variables denoting whether respondents were “very much” concerned about antisemitism in that context. Political ideology was measured by a question asking respondents whether they identified as liberal, moderate, or conservative. In order to control for the differences in Jewish background, current Jewish behavior, and connection with Israel, which Rebhun (2014) finds are associated with both experiences and perceptions of antisemitism, we developed three separate indices of childhood Jewish background, current Jewish behaviors, and “closeness to Israel,” each based on responses to a number of separate questions.^7^ Models also included controls for age at the time of survey (as a binary variable denoting whether respondents were aged 22 or older) and gender.^8^

To track overall trends in antisemitism during this period, we analyzed weighted estimates of the proportion of respondents who reported experiencing or being concerned about antisemitism at each time point. Trends in experiences of antisemitism are reported separately for undergraduates and non-undergraduates. For perceptions of antisemitism, we looked at the entire sample and separately reported trends in concern about antisemitism in the US and on college campuses. After reporting overall trends, we used binary logit models to identify factors associated with experiences and perceptions of antisemitism.

In addition to the independent variables discussed above, these models estimated the effect of time using a series of dummy variables for each time point, with March 2017 (the earliest time point) treated as the omitted category. Models of concern about antisemitism, either in the United States or on campus, also included an additional control for personally experiencing antisemitism. To estimate whether an independent variable, such as political ideology or student status, affected not only levels but also *trends* in antisemitism, we included interaction terms between the variable in question and the dummy variables for time.^9^ For key outcomes, the results of these models are presented using predicted probabilities, which present the model’s estimation of the likelihood that a respondent with a given set of characteristics will have a particular value for the dependent variable in question.

## Results

**Fig. 1:**
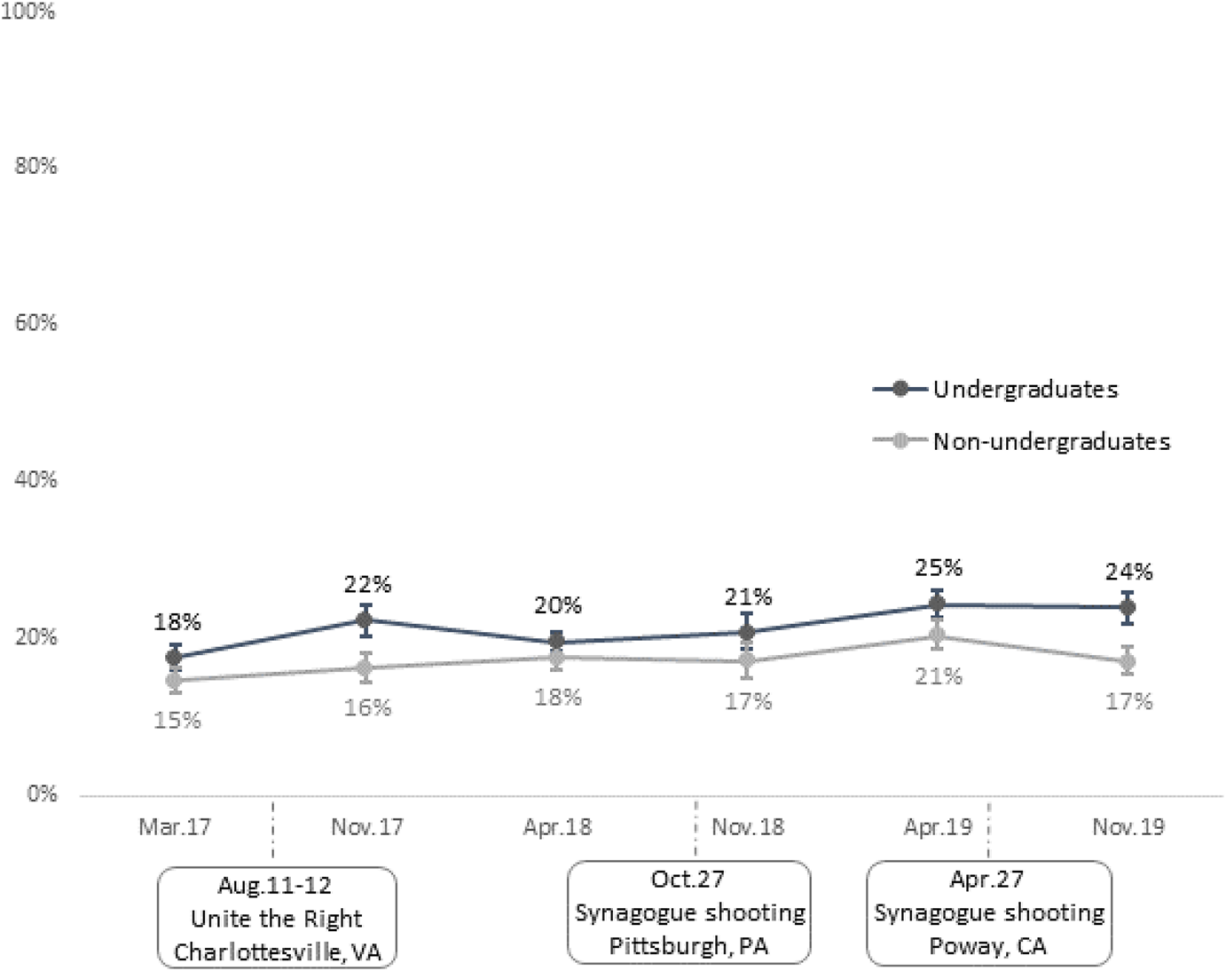
Any experience of antisemitic harassment by time. *Note*: Weighted proportions. Error bars denote 95% confidence intervals

Figure 1 presents overall trends in the proportion of our sample of Birthright applicants who experienced any form of antisemitic harassment between 2017 and 2019. The figure separates the findings for undergraduates and non-undergraduates. All figures also note the timing of key antisemitic events. Overall, undergraduates reported slightly higher rates of antisemitic harassment at each time point. Among undergraduates, we also see a small, temporary increase in experiences of antisemitism in the months following Charlottesville, and then a small, gradual increase over the next year and a half. Overall, the proportion of undergraduates who reported experiencing any form of antisemitic harassment in the past three months increased from 18% in March of 2017 to 24% in November of 2019. Among non-undergraduates, the rate of increase was less uniform during this period, with the proportion of young adults experiencing antisemitic harassment growing from 15% in March of 2017 to a high of 21% in April 2019, followed by a decline to 17% in November of 2019.

To confirm that the differences between undergraduates and non-undergraduates shown in Fig. 1 are not due to the influence of confounding variables, Table 2 presents results from a binary logit model of experiencing antisemitic harassment, controlling for undergraduate student status and other variables found to be associated with experiencing antisemitism. In this model, the coefficient for undergraduate student status remains positive and significant (*p* < 0.01). The model also indicates that Jewish young adults with higher levels of connection to Israel were more likely to experience antisemitism, and those with more Jewish friends were less likely to experience it. Furthermore, the model suggests that these characteristics have a far greater impact on the likelihood of experiencing antisemitism than being an undergraduate. Jews who identified as politically conservative were also more likely to experience antisemitism during this period than Jews who identified as politically liberal. The model also confirms that antisemitic experiences were significantly more likely to occur in April 2018 and April 2019 compared to the baseline of March 2017.^10^

**Table 2:** Binary logit model of experiencing any form of antisemitic harassment

	Coefficient	SE
Age 22–26	−0.064	(0.061)
Gender (female)	−0.160**	(0.045)
Undergrad student	0.165**	(0.058)
Both parents are Jewish	−0.240**	(0.054)
Political views (moderate)	0.037	(0.052)
Political views (conservative)	0.211**	(0.061)
Childhood Jewish background—low	−0.100	(0.061)
Childhood Jewish background—high	0.022	(0.057)
Number of J Friends—some/half	−0.365**	(0.051)
Number of J Friends—most	−0.677**	(0.069)
Number of J Friends—all	−1.353**	(0.116)
Current Jewish behavior index	0.228**	(0.027)
Israeliness index	0.288**	(0.027)
Time point—Nov.17	0.086	(0.063)
Time point—Apr.18	0.200**	(0.054)
Time point—Nov.18	−0.050	(0.074)
Time point—Apr.19	0.428**	(0.058)
Time point—Nov.19	0.095	(0.064)
Constant	−1.858**	(0.104)
Observations	25,696	

Robust standard errors clustered by individuals in parentheses. **p < 0.01; *p < 0.05

**Fig. 2:**
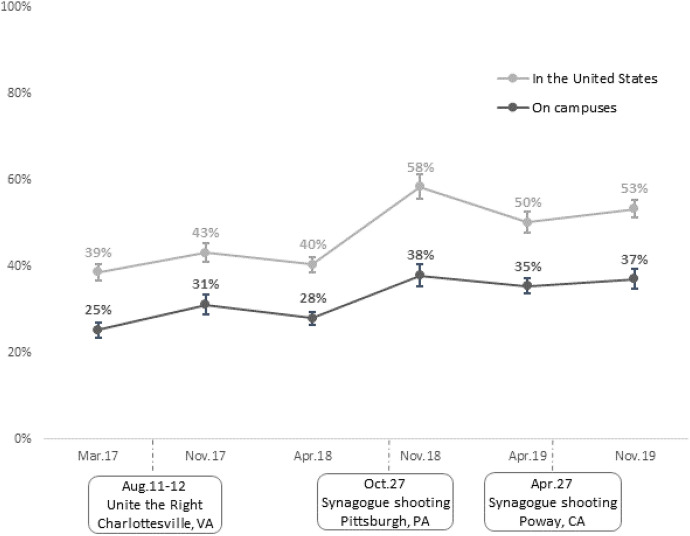
“Very much” concerned about antisemitism by time. *Note*: Weighted proportions. Error bars denote 95% confidence intervals

Regarding perceptions of antisemitism, Fig. 2 shows the proportion of Birthright applicants who were “very much” concerned about antisemitism in the United States, and the percentage who were “very much” concerned about antisemitism on college campuses, between March 2017 and November 2019. Overall, Jewish young adults were more concerned about antisemitism in the United States than on college campuses at each time point. In both contexts, there is a generally upward trend: between March of 2017 and November of 2019, the proportion of Birthright applicants who were “very much” concerned about antisemitism in the United States grew from 39 to 53%; during the same period the percentage who were “very much” concerned about antisemitism on campus increased from 25 to 37%. A dramatic, albeit temporary, spike in concern about antisemitism in the United States can also be seen in the month after the Pittsburgh shooting. Much smaller increases in concern about antisemitism in the United States and on college campuses are also evident in the months after the Unite the Right rally in Charlottesville and the shooting in Poway.

To explore the extent to which these results are influenced by political ideology, binary logit models were specified predicting the likelihood of expressing concern about antisemitism in the United States or on college campuses. For each context, we first present a model including controls for political ideology and the other covariates included in the model of experience above, as well as a control for whether respondents experienced any form of antisemitic harassment. We then specify a second model that interacts political ideology with the time dummies to test whether changes in concerns about antisemitism are significantly different for respondents of different political views.

Table 3 presents results from the model of concern about antisemitism in the United States. Model 1 shows that, as predicted, moderates and conservatives were significantly less concerned about antisemitism in the United States compared to liberals, after adjusting for other demographic and Jewish background characteristics. The model also indicates that, as expected, experiencing antisemitism was associated with greater levels of concern about antisemitism, and that concerns about antisemitism in the United States were higher in 2018 and 2019 than they were in 2017. Model 2, which adds interaction terms between ideology and time, shows that the increase in concern about antisemitism in November 2018—a month after the Pittsburgh shooting—was significantly smaller for conservatives than for liberals.

**Table 3:** Binary logit model of concern about antisemitism in United States

	Model 1		Model 2	
Variables	Coefficient	SE	Coefficient	SE
Age 22–26	0.167**	(0.046)	0.170**	(0.046)
Gender (female)	0.534**	(0.037)	0.534**	(0.037)
Undergrad student	−0.006	(0.044)	−0.002	(0.044)
Both parents are Jewish	−0.065	(0.043)	−0.067	(0.043)
Political views (moderate)	−0.512**	(0.044)	−0.470**	(0.087)
Political views (conservative)	−0.600**	(0.053)	−0.454**	(0.095)
Childhood Jewish background—low	0.006	(0.048)	0.005	(0.048)
Childhood Jewish background—high	−0.099*	(0.046)	−0.098*	(0.046)
Number of J Friends—some/half	0.060	(0.042)	0.061	(0.042)
Number of J Friends—most	0.249**	(0.056)	0.251**	(0.056)
Number of J Friends—all	0.064	(0.090)	0.058	(0.090)
Current Jewish behavior index	0.049*	(0.023)	0.050*	(0.023)
Israeliness index	0.324**	(0.021)	0.323**	(0.021)
Experienced antisemitism	0.849**	(0.044)	0.849**	(0.044)
Time point—Nov.17	0.001	(0.050)	0.000	(0.061)
Time point—Apr.18	0.132**	(0.043)	0.136*	(0.053)
Time point—Nov.18	0.658**	(0.059)	0.757**	(0.074)
Time point—Apr.19	0.498**	(0.047)	0.536**	(0.059)
Time point—Nov.19	0.430**	(0.050)	0.475**	(0.062)
Moderate × Nov.17	–	–	0.036	(0.134)
Moderate × Apr.18	–	–	0.012	(0.111)
Moderate × Nov.18	–	–	−0.116	(0.146)
Moderate × Apr.19	–	–	−0.094	(0.121)
Moderate × Nov.19	–	–	−0.064	(0.124)
Conservative × Nov.17	–	–	−0.027	(0.144)
Conservative × Apr.18	–	–	−0.044	(0.123)
Conservative × Nov.18	–	–	−0.436**	(0.159)
Conservative × Apr.19	–	–	−0.116	(0.132)
Conservative × Nov.19	–	–	−0.195	(0.135)
Constant	−1.484**	(0.081)	−1.519**	(0.084)
Observations	25,602		25,602	

Robust standard errors clustered by individuals in parentheses. **p < 0.01; *p < 0.05

**Fig. 3:**
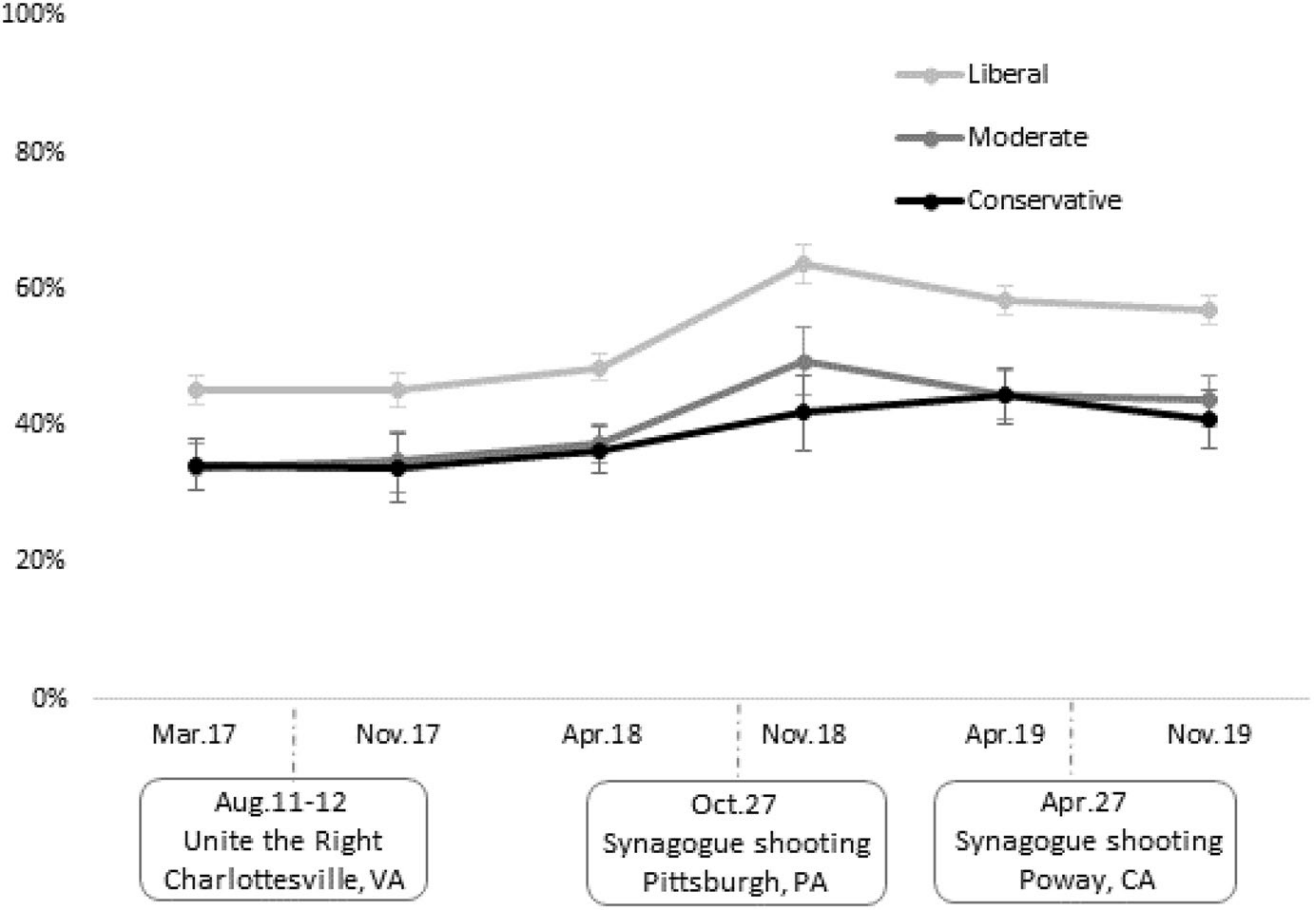
Predicted probabilities from the model of concern about antisemitism in the United States by ideology and time. *Note*: Predicted probability of being “very much” concerned about antisemitism in the US, derived from Model 2 in Table 3. Error bars denote 95% confidence intervals

To illustrate the magnitude of the effect of ideology and the dynamics of the interactions, Fig. 3 presents predicted probabilities of being “very much” concerned about antisemitism in the United States by ideology and time, holding other factors constant, derived from Model 2 in Table 3 above.^11^ These predictions indicate that at all time points, Jews who identified as liberals were significantly more concerned about antisemitism in the United States than moderate or conservative Jews with identical background characteristics. Moderates and conservatives appeared to have similar levels of concern about antisemitism at each time point, with one exception. The temporary spike in concerns about antisemitism in November of 2018, in the aftermath of the Pittsburgh shooting, was evident for liberal and moderate Jews, but not for conservatives.

Table 4 shows results from comparable binary logit models of concerns about antisemitism on college campuses. As before, Model 1 includes only main effects, while Model 2 adds interaction terms between political ideology and time. Model 1 shows that, contrary to our hypothesis, conservative Jews were not significantly more concerned about antisemitism on campus than liberals. Rather, political moderates were significantly less concerned than both liberals and conservatives. As expected, Model 1 also shows that respondents who were themselves undergraduates were significantly more concerned about antisemitism on college campuses than were non-undergraduates, although this relationship becomes nonsignificant when interaction terms are added. Results for other control variables are similar to those presented in Table 3 above. The interaction terms suggest that the difference in concern between liberals and conservatives was larger in November 2017 than it was in March of 2017, but is otherwise nonsignificant.

**Table 4:** Binary logit models of concern about antisemitism on campus

	Model 1		Model 2	
	Coefficient	SE	Coefficient	SE
Age 22–26	0.162**	(0.050)	0.162**	(0.050)
Gender (female)	0.389**	(0.040)	0.389**	(0.040)
Undergrad student	0.098*	(0.048)	0.094	(0.048)
Both parents are Jewish	−0.014	(0.046)	−0.017	(0.046)
Political views (moderate)	−0.212**	(0.046)	−0.290**	(0.100)
Political views (conservative)	0.070	(0.054)	0.182	(0.101)
Childhood Jewish background—low	0.019	(0.052)	0.021	(0.052)
Childhood Jewish background—high	−0.102*	(0.048)	−0.100*	(0.048)
Number of J Friends—some/half	0.113*	(0.046)	0.113*	(0.046)
Number of J Friends—most	0.385**	(0.058)	0.387**	(0.058)
Number of J Friends—all	0.245**	(0.094)	0.246**	(0.094)
Current Jewish behavior index	0.109**	(0.023)	0.110**	(0.023)
Israeliness index	0.486**	(0.023)	0.489**	(0.023)
Experienced antisemitism	0.874**	(0.043)	0.876**	(0.043)
Time point—Nov.17	0.032	(0.055)	−0.072	(0.070)
Time point—Apr.18	0.194**	(0.049)	0.201**	(0.062)
Time point—Nov.18	0.306**	(0.063)	0.312**	(0.078)
Time point—Apr.19	0.526**	(0.052)	0.560**	(0.067)
Time point—Nov.19	0.298**	(0.054)	0.343**	(0.068)
Moderate × Nov.17	–	–	0.282	(0.147)
Moderate × Apr.18	–	–	0.151	(0.127)
Moderate × Nov.18	–	–	0.129	(0.158)
Moderate × Apr.19	–	–	0.105	(0.136)
Moderate × Nov.19	–	–	−0.045	(0.136)
Conservative × Nov.17	–	–	0.283*	(0.144)
Conservative × Apr.18	–	–	−0.186	(0.129)
Conservative × Nov.18	–	–	−0.165	(0.165)
Conservative × Apr.19	–	–	−0.270	(0.139)
Conservative × Nov.19	–	–	−0.187	(0.139)
Constant	−2.798**	(0.093)	−2.811**	(0.096)
Observations	25,625		25,625	

Robust standard errors clustered by individuals in parentheses. **p < 0.01, *p < 0.05

**Fig. 4:**
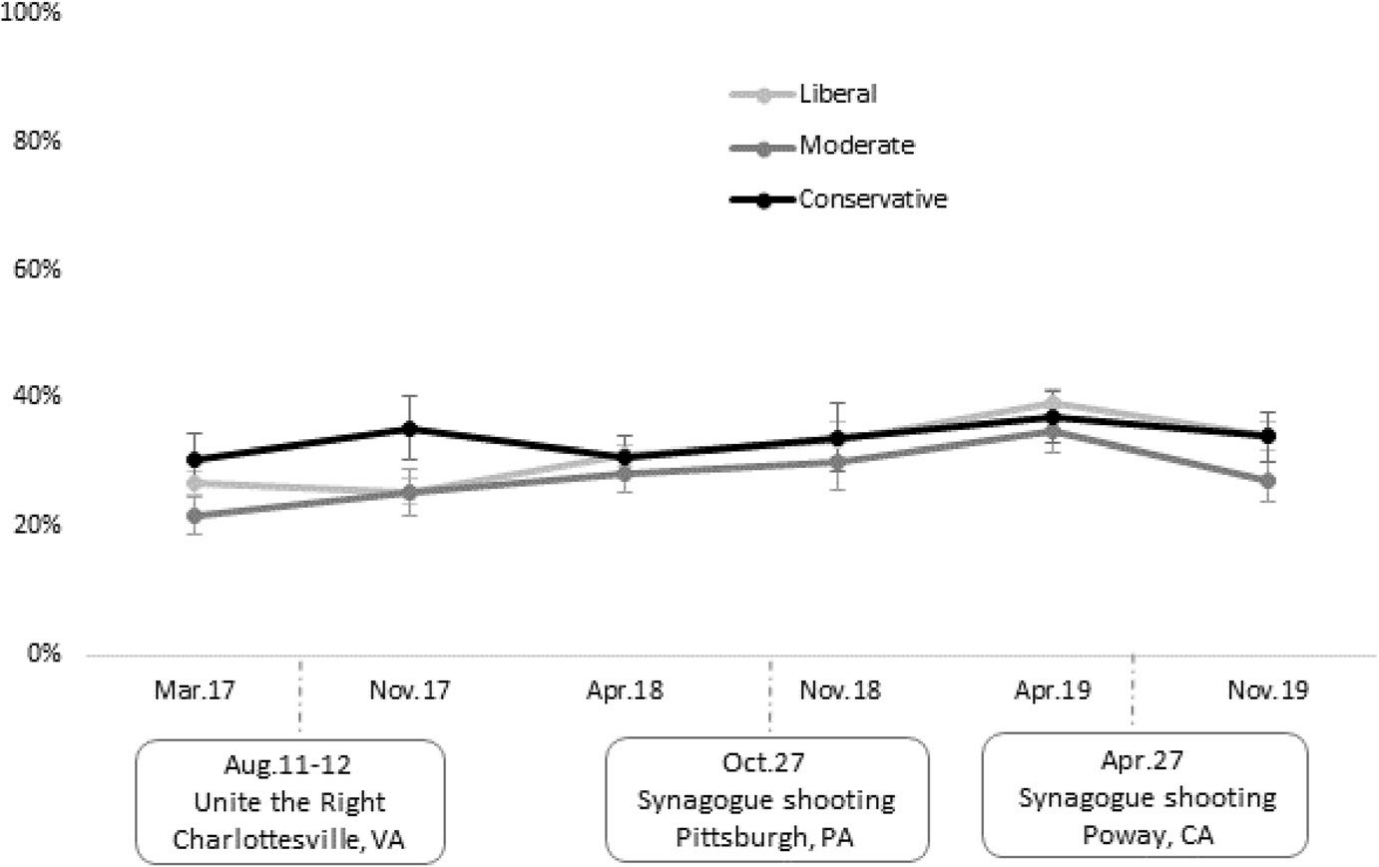
Predicted probability of concern for antisemitism on campus by ideology and time. *Note*: Predicted probability of being “very much” concerned about antisemitism in the college campus, derived from Model 2 in Table 4. Error bars denote 95% confidence intervals

To illustrate the dynamics of this relationship, Fig. 4 shows predicted probabilities of being “very much” concerned about antisemitism on campus by ideology and time, based on the results of Model 2 in Table 4. There appear to be few differences in the overall trajectory of concerns about antisemitism on college campuses by ideology, at least since 2018. As implied by the significant interaction term, the 2017 estimates suggest that conservative Jews were indeed more concerned about antisemitism than liberal or moderate Jews. Since 2018, however, liberal, moderate, and conservative Jews appear to have almost an identical likelihood of being “very much” concerned about antisemitism on college campuses: Among all three groups, concern grew gradually through 2018 and the first few months of 2019 before declining slightly in November 2019.

## Discussion

These data suggest that between 2017 and 2019, there was no dramatic rise in the rate at which Jewish young adults experienced antisemitic harassment—either on or off campus. Overall, Jewish undergraduates were more likely to experience antisemitic harassment than their peers in off-campus settings, especially in the second half of 2019. These differences, while statistically significant, are relatively small. The results confirm previous findings that, although a small number of schools may be “hotspots” of antisemitism, many Jewish undergraduates perceive little or no antisemitism on their campus (Saxe et al. 2016; Wright et al. 2017). Although our investigation is focused on younger Jews, it seems difficult to imagine that a dramatic surge in antisemitic harassment could exist in the United States without also being evident among Jewish young adults.

During this period, however, a substantial increase in Jewish young adult *concern* about antisemitism in the United States generally and on college campuses in particular does seem evident. Overall trends suggest that the Tree of Life shooting in Pittsburgh, in particular, was associated with elevated concerns over antisemitism in subsequent months. Increases in concern about antisemitism in the months after Charlottesville and Poway were much smaller but were part of a general upward trend in Jewish young adult concern about antisemitism during this period.

A complicated relationship appears to exist between political ideology and perceptions and experiences of antisemitism during this period. We find that conservative Jews are more likely to report experiences of antisemitism, even after controlling for Jewish background and connection to Israel, compared to liberal Jews. One possible explanation of this result is that Jewish conservatives may be more likely than liberals to see harsh criticisms of Israel as instances of antisemitism, due to Jewish conservatives’ lower tolerance for criticism of Israel for any reason (Wright et al. 2020). Despite this finding, and contrary to our expectation, no evidence was found of an ideological divide in Jewish concerns about antisemitism on campus. Although there is some evidence that conservative Jews may have been slightly more concerned about antisemitism on campus in 2017, this effect seems to have disappeared by 2018. By 2019, liberal and conservative Jews were almost equally likely to be concerned about antisemitism on campus. Perhaps the absence of deep ideological divisions regarding antisemitism on campuses stems from the fact that antisemitism on campus is driven by criticism of Israel *and* traditional anti-Jewish stereotypes (Saxe et al. 2016; Wright et al. 2019a, b). Thus, notwithstanding the politicized tenor of media discussions about this issue, there appears to be no simple relationship between political ideology and the challenges of antisemitism on campus.

There is, however, clear evidence of an ideological divide in Jewish young adults’ perceptions of antisemitism in the United States. Despite the fact that Jewish conservatives were most likely to report experiences of antisemitism, Jews who identified as liberal were significantly more concerned about antisemitism in the United States than were their moderate and conservative peers. We also find ideological differences with respect to *trends* in concern about antisemitism in the United States: the spike in concern after Pittsburgh was evident for liberal and moderate Jewish young adults, but not for conservatives. These differences may be partly due to differences in the foci of liberal and conservative media discourse surrounding antisemitism. Future studies should continue to examine political ideology in relation to Jewish perceptions of antisemitism.^12^

That liberal Jews became far more concerned about antisemitism in the United States during a period in which a Republican president has been repeatedly accused of stoking bigotry and discrimination towards Jews, Blacks, immigrants, and other marginalized groups (Sides, Tesler, and Vavreck 2018; Graham et al. 2019) supports earlier findings that Jewish concerns about antisemitism are linked to broader feelings about the climate for marginalized populations in America (Cohen 2018). Liberal Jews’ growing concerns about antisemitism during the Trump era may partly reflect a profound dissatisfaction with racial injustice during this period. Jewish commentators have occasionally expressed concerns about the Black Lives Matter movement’s ties to the BDS movement and the movements’ occasional reference to antisemitic tropes and stereotypes (Green 2016). But these findings and those of Cohen (2018) suggest that the rise of the Black Lives Matter movement may have actually acted to increase Jewish concerns over antisemitism (at least among liberal Jews) not because the movement itself contributes to antisemitism, but because it serves as a reminder that American society is often still hostile and unwelcoming to members of ethnic and religious minority groups. Images of violence and discrimination against people of color in America may, for some Jews, invoke Martin Niemöller’s famous confession: “First they came for the socialists, and I did not speak out—because I was not a socialist….”

## Conclusion

At first glance, these data seem to suggest dissonance between young adult Jews’ concerns about antisemitism and their personal experiences of antisemitic harassment. Despite the troubling events of 2017–19, there has been little increase in the prevalence of antisemitic experiences among Jewish young adults during this period. Yet, since 2017, Jewish young adults have nevertheless become dramatically more concerned about antisemitism in the United States and on college campuses. If young Jews in 2019 were no more likely to personally experience antisemitism than they were in 2017, then why were they so much more concerned about it?

One possibility is that, given the history of antisemitism and the alarming frequency of mass shootings in America during this period, Jews have been primed to see even rare events like the murders in Poway and Pittsburgh not as outliers or isolated incidents, but as harbingers of future violence. Since 2017, the shootings in Parkland, El Paso, Las Vegas, and elsewhere have made clear that individuals—including white nationalists—who wish to commit mass murder in America have few difficulties acquiring the means to do so. In an environment where virulent antisemitic conspiracy theories are widely disseminated on social media, it should not be surprising that Jewish young adults are becoming more concerned about their own safety, even if they have no firsthand experiences of antisemitism.

At the same time, Jewish young adults’ concerns about antisemitism may reflect more than anxieties about violence. Especially for young Jews who never before thought of themselves as an “other” in American society, hearing antisemitic language on social media and on campus may trigger apprehension about the extent to which they are “welcome” in their own community—be it their campus, city, state, or country. Should they hide their Jewish identity in conversations with friends or classmates? Will they be attacked on social media for criticizing antisemitism? Will they be dragged into contentious debates about Israel simply because they are Jewish?

Understanding the nature of what appears to be dramatic growth in American Jews’ concerns about antisemitism in America will require further investigation. Enumerations of antisemitic “incidents” provide little insight on this issue—the focus of efforts to understand this phenomenon must be Jews themselves. Of course, the myriad effects of the COVID-19 pandemic, continued protests against racial injustice and police violence, and the results of the 2020 US presidential election are likely to profoundly affect developments described here in ways that are difficult to predict. Future work tracking these trends will be necessary to provide up-to-date analyses of Jewish perceptions of and experiences with antisemitism.

## Supplementary Information

Below is the link to the supplementary information.

Supplementary material 1 (DOCX 67 kb)

## Footnotes

1 Although our focus is on understanding national-level trends, other recent research suggests that geographic location may also impact both perceptions and experiences of antisemitism in complex ways. For example, Cohen (2010) finds that Jewish concerns about antisemitism are independently influenced by both the number of antisemitic incidents in that state and the frequency of reports about antisemitic incidents in local media. Feinberg (2020) finds that antisemitic incidents themselves are more common in states with larger Jewish populations, but also in states with larger numbers of hate groups. Although beyond the scope of this paper, future work should explore how these geographical factors may impact the national-level trends analyzed here.

2 During this period, Birthright Israel’s eligibility criteria were broadened to include individuals between the ages of 27 and 32, but to preserve comparability across time points, we exclude these older applicants from all analyses. Canadian Birthright applicants (who were included in the original surveys) are also excluded from all analyses.

3 Analysis revealed that participation in Birthright Israel had no substantial or consistent impact on any of the dependent variables analyzed in this paper. However, in order to ensure that Birthright participation did not bias results in any way, all analyses presented here were replicated on a subsample of those who did not participate in the trip itself. Due to the loss in statistical power, these analyses had larger standard errors than those reported here but produced substantively similar results in all cases. To show that the dynamics reported here were not substantively impacted by Birthright participation, Supplementary Appendix 4 presents estimates of perceptions and experiences of antisemitism over time, separately for the full population of respondents analyzed in this paper, and for the subpopulation of applicants who never participated in Birthright.

4 The Jewish background characteristics of Birthright applicants included in this study are not substantially different from those of the broader population of American Jewish young adults (Wright, Hecht, and Saxe 2019a, b, 39). Critically, the applicant pool contains individuals from highly religious Jewish families, including many individuals who had already been to Israel prior to applying to Birthright, as well as those with almost no prior connections to Jewish life.

5 In particular, we weighted on the following variables: gender, age, Jewish education, past visits to Israel, having two Jewish parents, and participation in a Birthright trip. These data were available for all eligible applicants (including survey non-respondents), and thus respondents were weighted to the full population of summer 2017 applicants. These weights therefore correct for any nonresponse bias stemming from differential nonresponse with respect to these variables. It is, of course, possible that differential nonresponse correlated with other variables could have biased results. However, an experimental study found very low levels of nonresponse bias—even on outcome variables related to Jewish life—in similar surveys of Birthright applicants that had response rates as low as 10% (Wright 2015).

6 Respondents who were not themselves college students were asked how concerned they were about antisemitism on “college campuses,” while those who were currently college students were asked about “college campuses in general” to clarify that the question was not asking about antisemitism only on their own campus.

7 See Supplementary Appendix 1 for details of index construction and descriptive statistics for all variables.

8 We confirmed that this age variable is not collinear with undergraduate status (see Supplementary Appendix 2).

9 Because many individuals responded to both the pre- and post-trip survey in each year, cluster robust standard errors are used in all models to account for the nesting of observations within individuals.

10 We also ran a comparable model that included an interaction between student status and time. Although the interaction terms are nonsignificant, the model broadly confirms the results presented in Fig. 1; the difference between students and non-students is most pronounced in November 2019 and almost nonexistent in 2018 (see Supplementary Appendix 3).

11 In these and other predictions discussed in this paper, we hold all other variables at their mean values.

12 Although it was not a focus of our research, our results also point to a complex gender dynamic. Females were significantly less likely to *experience* antisemitism. However, after accounting for gender differences in experience of antisemitism (and political ideology), females appeared significantly more *concerned* about antisemitism (both on the college campus and in the US in general). A detailed analysis of this relationship is beyond the scope of this paper, but future work should explore the role of gender in Jewish perceptions and experiences of antisemitism.
