# Supplementary material for: Trends in Jewish Young Adult Experiences and Perceptions of Antisemitism in America from 2017 to 2019
Source: Contemp Jew. 2021 Feb 15;41(2):461–81. doi: 10.1007/s12397-021-09354-6 (PMC7883958; doi:10.1007/s12397-021-09354-6)
Supplement: Supplementary file 1 — Supplementary material 1 (DOCX 67 kb) [file 12397_2021_9354_MOESM1_ESM.docx]

**Supplementary Appendices for “Antisemitism: Here and Now? Trends in Jewish Young Adult Experiences and Perceptions of Antisemitism in America from 2017 to 2019”**

**Appendix 1: Details of Index Construction and Variable Descriptions**

**Jewish background index**

This index was created in four steps:

Step 1: Using the following six variables we count the number of Jewish experiences a respondent had in childhood

- Celebrated Hanukkah
- Celebrated Seder
- Attendance Jewish religious services
- The family kept kosher at home
- Having Shabbat meal
- Celebrated a bar/bat mitzvah

Step 2: We identify the most advanced level of Jewish education for each respondent

- Jewish Day school
- Supplementary Jewish education
- None

Step 3: We identify whether a respondent had any of the following informal Jewish experiences

- Attended a Jewish day camp
- Attended a Jewish overnight camp
- Was a member of a Jewish youth group

Step 4: The variables created in Steps 1-3 were z-scored and summed. The resulting variables were then recoded into “Low,” “Medium and “High” categories based on the resulting distribution.

**Current Jewish behavior**

We used factor analysis to create an index of current Jewish behaviors out of the following variables

- Having Shabbat meal
- Belonging to the orthodox denomination
- Attendance of Jewish religious services
- Participation in a social event sponsored by a Jewish organization

**Israeliness index**

We used factor analysis to create an index out of the following variables

- Connection to Israel
- Emotional attachment to Israel
- Confidence in understanding the situation in Israel
- Number of visits to Israel

Table 5: Descriptive Statistics

|  |  | % | N |
| --- | --- | --- | --- |
| Experienced antisemitism | No experience of antisemitism | 80% | 23,198 |
|  | Experienced antisemitism | 20% | 5,466 |
|  |  |  |  |
| Concerns about antisemitism on campuses | 1. Not at all | 13% | 3,823 |
|  | 2. A little | 23% | 6,500 |
|  | 3. Somewhat | 32% | 9,095 |
|  | 4. Very much | 33% | 8,993 |
|  |  |  |  |
| Concerns about antisemitism in your country | 1. Not at all | 4% | 1,151 |
|  | 2. A little | 14% | 4,265 |
|  | 3. Somewhat | 34% | 9,697 |
|  | 4. Very much | 48% | 13,284 |
|  |  |  |  |
| Political views | Liberal | 62% | 17,475 |
|  | Moderate | 21% | 5,848 |
|  | Conservative | 17% | 4,542 |
|  |  |  |  |
| Jewish childhood experience index | Low | 23% | 7,874 |
|  | Medium | 49% | 15,035 |
|  | High | 28% | 8,310 |
|  |  |  |  |
| Number of Jewish friends | None/hardly any | 24% | 7,423 |
|  | Some/Half | 49% | 14,322 |
|  | Most | 19% | 5,646 |
|  | All | 8% | 2,218 |
|  |  |  |  |
| Jewish parents | One parent or None | 33% | 12,139 |
|  | Both parents are Jewish | 67% | 20,250 |
|  |  |  |  |
| Age group | 18-21 | 49% | 18,207 |
|  | 27-32 | 51% | 14,389 |
|  |  |  |  |
| Gender | Female | 57% | 21,328 |
|  |  |  |  |
| Student status | Being Undergraduate student | 50% | 17,635 |
|  |  | Mean | Std.Dev. |
| Index for current Jewish behavior | | 0.89 | 1.03 |
| Israeliness Index | | 2.22 | 0.1 |

**Appendix 2: Multicollinearity Analysis**

Table 5 presents Variance Inflation Factors (VIF) for the independent variables used in all models presented in the paper to assess the level of multicollinearity among the independent variables. FIV scores for all variables are below 3, and in most cases well below 2, indicating relatively low multicollinearity for the reported models.

Table 6: Variance Inflation Factors for independent variables used in reported models

| Variable | VIF | Square root of VIF | Tolerance | R-Squared |
| --- | --- | --- | --- | --- |
| Age (>22) | 2.03 | 1.42 | 0.4929 | 0.5071 |
| Gender | 1.04 | 1.02 | 0.9599 | 0.0401 |
| Undergraduate | 1.87 | 1.37 | 0.5345 | 0.4655 |
| Having to Jewish parents | 1.42 | 1.19 | 0.7055 | 0.2945 |
| Political views | 1.18 | 1.09 | 0.8491 | 0.1509 |
| Jewish childhood index | 1.54 | 1.24 | 0.651 | 0.349 |
| Number of Jewish friends | 1.48 | 1.22 | 0.6736 | 0.3264 |
| Current Jewish behavior index | 1.66 | 1.29 | 0.6039 | 0.3961 |
| Israel index | 1.3 | 1.14 | 0.7667 | 0.2333 |
| Experienced antisemitism | 1.03 | 1.02 | 0.9699 | 0.0301 |

**Appendix 3: Alternative Analysis of Experiencing Antisemitism with Interaction between Student Status and Time**

Table 7: Binary logistic regression of experiencing antisemitism with the interaction between student status and time

|  | All | |
| --- | --- | --- |
| VARIABLES | Coef. | SE |
| Age 22-26 | -0.055 | (0.061) |
| Gender (Female) | -0.160** | (0.045) |
| Undergrad student | 0.116 | (0.094) |
| Both parents are Jewish | -0.239** | (0.054) |
| Political views (moderate) | 0.037 | (0.052) |
| Political views (Conservative) | 0.210** | (0.061) |
| Childhood Jewish background - low | -0.100 | (0.061) |
| Childhood Jewish background - High | 0.020 | (0.057) |
| Number of J Friends - Some/Half | -0.363** | (0.051) |
| Number of J Friends - Most | -0.676** | (0.069) |
| Number of J Friends - All | -1.355** | (0.116) |
| Current Jewish behavior index | 0.227** | (0.027) |
| Israeliness index | 0.289** | (0.027) |
| Time point - Nov.17 | -0.016 | (0.096) |
| Time point - Apr.18 | 0.241** | (0.086) |
| Time point - Nov.18 | -0.004 | (0.113) |
| Time point - Apr.19 | 0.408** | (0.092) |
| Time point - Nov.19 | 0.015 | (0.096) |
| Undergrad x Nov.17 | 0.176 | (0.129) |
| Undergrad x Apr.18 | -0.067 | (0.111) |
| Undergrad x Nov.18 | -0.086 | (0.146) |
| Undergrad x Apr.19 | 0.031 | (0.118) |
| Undergrad x Nov.19 | 0.148 | (0.125) |
| Constant | -1.834** | (0.111) |
|  |  |  |
| Observations | 25,696 |  |

*Note: Robust standard errors in parentheses. ** p<0.01, * p<0.05*

Figure 5: Predicted probability of experiencing antisemitism, derived from the model where student status is interacted with time.


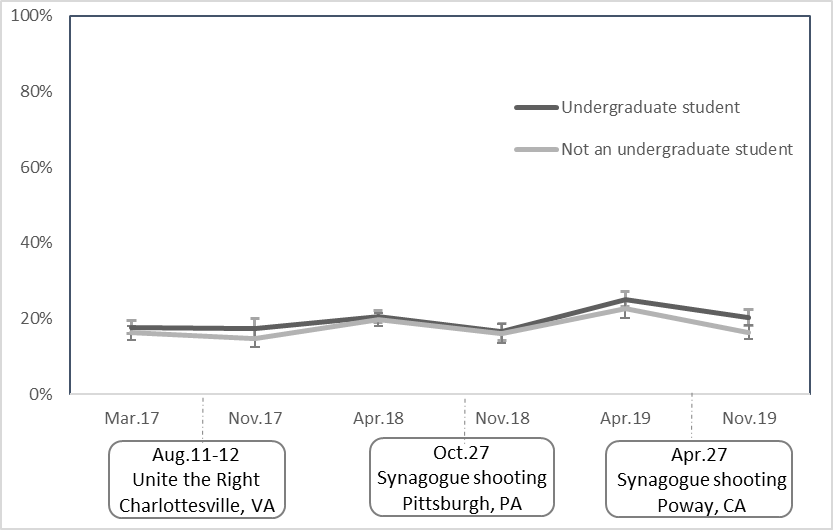


*Error bars denote 95% confidence intervals*

**Appendix 4: Alternative analyses limited to Birthright Nonparticipants**

Of the total sample of 32,596 observations collected across these six time points, 27,014 corrispond to applicants who went on a Birthright trip between the pre- and post-trip surveys, while 5,582 corrispond to applicants who never participated in a Birthright trip. The following charts present weighted proportions of experience and perceptions of antisemtism by time, seperatly for all respondents and limited to only those respondents who never participated in Birthright Israel.

Figure 6: Any experience of antisemitic harassment by time

Figure 7: “Very much” concerned about antisemitism by time

Figure 3: Concern about antisemitism on Campus
